# Patterns of social participation and impacts on memory among the older people

**DOI:** 10.3389/fpubh.2022.963215

**Published:** 2022-11-15

**Authors:** Hu Han, Zhang Hengyuan, Tang Yonggang

**Affiliations:** ^1^School of Public Policy and Administration, Xi'an Jiaotong University, Xi'an, China; ^2^General Office of the CPC Shaanxi Provincial Committee, Xi'an, China

**Keywords:** social participation, elderly people, lifestyle sustainability, latent class analysis (LCA), memory

## Abstract

This study employs latent class analysis to analyze the social participation patterns of elderly Chinese, as well as the impact of different social participation patterns on memory. According to the results, older persons exhibit four distinct social participation patterns. and senior citizens with a strong emphasis on entertainment had a better memory than those with a low participation level. Although there was no difference in urban elders' memory across the four social engagement patterns, the connection varied significantly between urban and rural seniors. As a result, it is suggested that the elderly's memory be improved by protecting their right to social involvement, enriching the style and content of social participation, and assuring the continuity of their social obligations.

## Background

As the number and life expectancy of elderly people increases, society pays increasing attention to their quality of life. In the late 1990s, the World Health Organization proposed the term “active aging,” which was followed by the formulation of the term “Health-Participation-Security,” indicating that encouraging elderlies to involve themselves in is a crucial way to actively respond to population aging and improve their quality of life. Participation in social activities is one of the major factors that influence the cognitive function of the elderly. Through social participation, seniors can more easily undergo smooth psychological transition and adapt to the process of aging, which aids in their acquisition of more social resources and psychological identity, as well as the strengthening of their bodily systems and mental activity, which enhances physical health and lowers the risk of dementia. Nevertheless, due to a lack of resources and insufficiently age-appropriate community construction, the social participation of the elderly is still not widely practiced in China at this time. Social isolation among the elderly is referred to be a “contemporary behavioral plague” and has developed into a serious public health concern.

Most studies focus on one type of social participation and quality of life, e.g., the effects of physical activity, mental activity, or recreational activity on the health of older adults, ignoring the relationship between activity and health among many types. Mandatory social obligations disappear after retirement and are replaced by volunteers, contract workers, members of interest groups, etc. Seniors are actively taking up significant roles in the family, such as taking care of a needy spouse in the position of a wife or husband, taking care of elderly parents in the role of a child, taking care of children in the role of a parent, and taking care of grandchildren in the role of a grandmother.

However, the time and resources for each role are limited, and many older people may struggle to make a choice between personal life and family care or sacrifice the quality of life for one role for the other. Furthermore, some may sacrifice their right to choose their lifestyle to care for their family, resulting in an imbalance between personal and family roles. There is a scarcity of research examining the relationship between the various roles elderly people undertake in their personal and familial lives. Significantly, the combination of different responsibilities in personal and family life may affect the breadth and depth of elderly people's relationships with society and their memory capacity. Will placing high expectations on the elderly's position in the family result in a mismatch between their personal and family lives? Does it impact the personal growth of the elderly, such as memory loss?

This study used survey data gathered by Xi'an Jiaotong University in the provinces of Zhejiang and Hubei in 2021 to address the problems raised above. It employed latent class analysis to evaluate the social participation patterns of the Chinese elderly and examined the impact of various social participation patterns on the elderly's memory.

## Chinese context

Since the reform and opening, China has changed tremendously from a “society of acquaintances” to a “society of strangers.” In the traditional acquaintance society, the “different mode” of the interaction circle consists of social relations such as families and clans. With the advent of urbanization and the acceleration of citizenship, the agricultural population has transferred from rural areas formed by acquaintances to urban areas created by strangers, resulting in the gradual personalization of the lives of a significant number of individuals. Rebuilding their life circle and integrating into communal life provide new challenges for the elderly who relocate to cities or dwell in communities after retirement. Simultaneously, aging and its associated health problems are getting more severe as age accelerates.

Retirement life for the elderly in China is essentially separated into personal style and family style, and as life expectancy increases, senior caregiver roles for elderly parents and disabled spouses are growing more prevalent. The implementation of the three-child policy and the underdeveloped system of childcare services for 0–3-year-olds has shifted the pressure of caring for children from families to the elderly. Especially under the traditional Chinese family orientation, the elderly tend to consciously assume intergenerational childcare responsibilities and are even willing to make personal sacrifices for their children.

## Literature review

### Patterns of social participation among elderly

In recent years, domestic and international research has begun to simultaneously explore various categories of activity participation and classify older people's social participation activities into various types. Based on data from the US Health and Retirement Survey, a prospective category model was used to categorize a variety of social activities of older adults into five categories: low, medium, high, economic, and physical activity (HRS). Urban senior adults' social involvement activities were classified into five categories: work, social, leisure, domestic, and general. Lili and Bin classified the social activities of senior adults into three categories, including low participation type, family care type, and high participation type, using data from the 2014 China Longitudinal Aging Social Survey (1). The research on the social participation patterns of aged people in China is still in its infancy.

### Social engagement patterns and cognitive memory

Numerous studies have demonstrated that the physical and mental health of the elderly is significantly correlated with their social participation and that social high-quality participation can improve the health of the elderly, protect them from memory loss and dementia (2), and slow down the decline in perceptual speed (3). Studies have shown that taking part in categories of social activities can slow down the brain's decline and lower the risk of dementia (4). Socializing is a great way for the elderly to feel better mentally and get help from their peers (5). Scholars also have different ideas about how social participation changes cognitive function over time. Chang et al. found that physical activity in midlife kept cognitive function and reduced or delayed the risk of dementia later in life. This suggests that social participation has a cumulative effect on cognitive function over time (6). A study by Ybarra et al. also found that 10 min of social participation can improve cognitive functioning in the short term and that different types of social participation have different effects on cognitive functioning. Haslam et al. found that social participation includes both group and individual participation. They also found that individual participation in multiple socially organized groups had the most impact. Glei et al. found in their Asian study that people who didn't take part in any social group activities had a 13% higher risk of cognitive impairment at 3 years than people who took part in one or two social group activities (7).

### Work-family balance theory and social role theory: Historical context

The study of work-family relationships has experienced three stages: conflict, promotion, and equilibrium. Primitive research mostly relied on “the severity perspective” and “the expansion enhancement perspective” as its primary theoretical foundations (8). “The severity perspective” holds that individual role resources are limited, and different roles constantly consume individual psychological and physiological resources, thereby weakening or even damaging individual functions (9). Work and family have additional requirements for individuals, so success in one field may be based on sacrifice in the other (10, 11). In contrast, the “reinforcement hypothesis” asserts that the involvement of multiple roles can make individuals obtain more happiness and gains, because individual power, reputation, resources, and emotional satisfaction have been strengthened (12–14).

As the study has progressed, it has been discovered that the interaction between work and family cannot accurately reflect their genuinet relationship from either a negative or positive perspective. Therefore, in order to better understand the relationship between the two fields, researchers put forward the concept of “work-family balance” from an integrative perspective. They believe that a certain role expectation reached by individuals through negotiation and sharing with their collaborators in the field of work and family is work-family balance (15–17). This definition measures work-family balance based on a person's duties and obligations in both areas. Studies have shown that individuals who can balance work and family roles face fewer conflicts, which is more conducive to improving their health level (18).

Role changes in later life will impact the health of the elderly. When people retire from the workforce, their social responsibilities shift, affecting their physical and mental health. The ability of the elderly to accept role adjustments in later life and seize the worth of their lives also influences their physical and psychological health. Some retirees who prioritize their families are content to enjoy their grandchildren and live a peaceful family life, whereas others prefer to remain active in the social, political, economic, and cultural arenas for economic or self-realization purposes. The shifting of roles is conceptualized differently by various perspectives. The activity theory proposes that the more active elderly individuals are, the greater their life satisfaction and the better their health. The separation theory claims that old age is different from middle age, and it's healthier for the elderly to be less active, less involved with others, and focus on their inner life. The continuum theory implies that each person seeks to maintain a coordinated pattern of activity, replacing lost responsibilities with similar ones to maintain physical and mental health.

**Figure 1:**
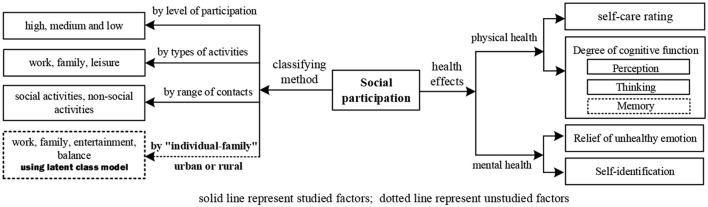
Research status of social participation.

Current studies analyze the social participation patterns of older adults by the level of participation, type of activities, and whether they are exposed to society, with more qualitative perspectives on the effects on older adults' disability and cognitive function, etc Very little research has explored the participation patterns of the elderly from an individual-family perspective (19). Rapid socioeconomic development has transformed the social role and family structure of the elderly, resulting in despair and anxiety, which affect their cognitive function. In order to minimize and delay cognitive decline, we should focus on social variables at an early stage (20, 21). “Prevention” is more important than “treatment,” so we should examine the factors specifically affecting elderly cognitive function (see the authors' drawing in Figure 1). Therefore, we analyzed domestic and international literature on the link between social factors and memory function and used survey data to investigate the interaction between memory function and different types of social involvement in the elderly from an individual-family viewpoint (the dotted line part in Figure 1).

## Research framework

**Figure 2:**
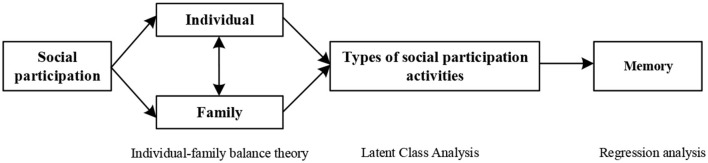
Analytical framework.

Based on the above theory, the long-standing divide between urban and rural areas in China has led to different patterns of social participation among the elderly in urban and rural areas. This may have different effects on their cognitive abilities, such as their memory. According to the literature review, this study focuses on three topics, (1) What are the social participation patterns of the elderly in terms of individual-family perspective? (2) What are the impacts of different participation patterns on the memory function of the elderly? (3) What's the difference between the effects on elderly persons in rural vs. urban areas? (Figure 2).

## Materials and methods

### Data sources

**Figure 3:**
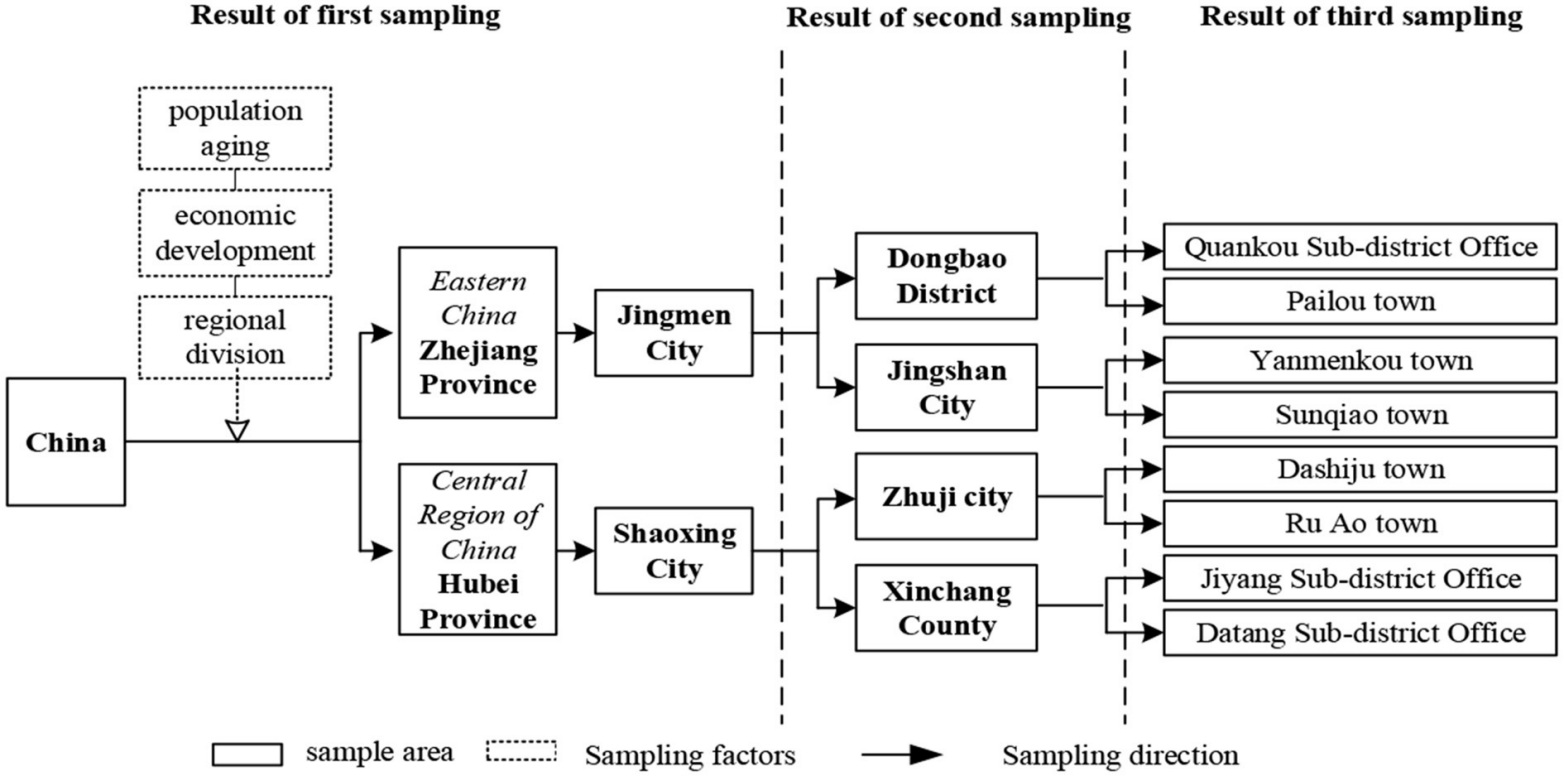
The sampling method of the CECS survey.

The survey data collected information on personal and family characteristics, social activities, services, and insurance of people aged 60 and above and was practiced in Hubei and Zhejiang provinces in 2021. The survey team was composed of 21 teachers, doctoral students, and master's students from Xi'an Jiaotong University. Based on the stratified random sampling method, Jingmen City in Hubei Province and Shaoxing City in Zhejiang Province were chosen as the first-layer samples. Dongbao District, Jingshan City of Jingmen City, and Zhuji City, Xinchang County of Shaoxing City, were selected as the second-layer samples. One sub-district or township and one pension institution from each District/City/County were chosen as the third-layer (Figure 3). We used the random sampling technique to choose about 125 old people aged 60 years and above in each third- layer. To ensure that all questions in the questionnaire were answered as completely as possible, the investigators directly asked old people questions and filled in the questionnaire accurately. Nine hundred forty-seven questionnaires were delivered in the provinces of Zhejiang and Hubei. After quality check and screening, we deleted samples that missed multiple key questions and 913 questionnaires were retained in the end. The questionnaire was funded by the Research Foundation of China's Ministry of Education (18JZD045), and its design adheres to the principles of rationality, precision, logic, non-induction, understandability, etc. The preliminary content has been discussed and modified by experts many times; this paper uses the questionnaire's basic personal information, social participation, and other topics.

All methods were carried out following relevant guidelines and regulations. Study protocols and consent forms were approved by the medical ethics committee of the Health Science Center of Xi'an Jiaotong University (approval number 2018–1200). All participants have the independent capacity for civil conduct and provided informed consent to participate in the study.

### Variables selection

The elderly's memory was defined as a dependent variable in our analysis for this study. Based on previous research, we evaluated the respondents' ability to recall events from the previous 3 days to determine their memory (22, 23). The question is “How many things can you remember in 3 days?” with a 5-point Likert scale. Participants were deemed to have an excellent memory of “Everything” or “Most things.” Participants were regarded to have memory impairments if their response was “Only important things,” “Very few things,” and “Nothing.” Although it is a single-item question, this is one of the most frequently used questions, because it has the advantage of being simple and fast, intuitive, and inexpensive to administer. If the participant is unwilling or unable to answer the question explicitly, we will remove his questionnaire from the sample.

Relevant studies classify the social participation activities of elderly adults based on multiple dimensions, including family, work, civil society, and social psychology (24). We employ six questions to gauge older individuals' participation, which is the data source for latent class analysis. First, family participation is measured by whether the elderly care for their grandchildren. If the participants are caring for grandchildren, they were considered with family participation. Second, we evaluate work participation by asking if retirees are employed and if they receive re-employment services. If the participants are doing paid work, we consider them as employed. And we asked participants whether they had received re-employment services after retirement to measure work participation further. Thirdly, in order to comprehend the participation of the elderly in civil society, we inquired as to whether or not they participated in recreational activities (such as sports or artistic activities) and volunteer activities after retirement. The elderly's social psychology is also measured by asking whether they obtain psychological counseling after retirement.

Referring to relevant research, demographic variables were used as control variables, including gender, age, living ability, and education. Age referred to the chronological age of the respondents when they are interviewed in 2021. Gender was grouped as male and female. Education was grouped as illiteracy, primary school, junior high school, and high school and above. Marital status was grouped as married and single, which included those who reported being divorced, widowed, and never married.

In summary, the measurements of variables are shown in Table 1.

**Table 1:** Measurements of valuables.

**Variable type**	**Variable name**	**Measurement format**	**Response options and value assignment**
Dependent variable	Memory	Remember what happened in the last 3 days	0 = bad, 1 = good
Independent variable	Care for grandchildren	Is taking care of their grandchildren	0 = no, 1 = yes
	Employed	Is doing paid work after retirement	0 = no, 1 = yes
	Re-employment service	Have received reemployment services after retirement	0 = no, 1 = yes
	Volunteer	Participate in voluntary activities after retirement	0 = no, 1 = yes
	Recreational activities	Participate in sports or artistic activities after retirement	0 = no, 1 = yes
	Psychological counseling	Have received psychological counseling after retirement	0 = no, 1 = yes
Control variable	Gender	Gender	0 = male, 1 = female
	Age	Age in 2021	value ≥60
	Disability	The evaluation result of the ADL scale is a disability	0 = no, 1 = yes
	Education	Level of education attained	1 = illiteracy, 2 = primary school, 3 = junior high school, 4 = high school and above
	Marital status	Marital status	0 = single, 1 = married

### Methods

First, means and frequency distribution were used to report individual characteristics of respondents, dependent variables, and independent variables. Next, the questions related to social participation were extracted to classify the social participation behavior of the elderly. Latent Class Analysis (LCA) was conducted to classify the social participation patterns of the elderly. χ^2^ test is used to analyze the differences between different social participation patterns. Finally, we examined the effect of the category of social participation on the memory of the elderly using binary logistic regression. In this study, SPSS version 20.0 (IBM Corp., Armonk, USA) was used in frequency distribution and binary logistic regression. Mplus 8.3 (Linda Muthén & Bengt Muthén, USA) was used in latent class analysis.

## Results

### Descriptive statistics

Descriptive statistics of variables are shown in Table 2. 59.04% of the respondents live in the urban area, and 40.96% of them live in rural areas. The majority of the respondents were female (54.49) and married (72.71%). 81.69% of respondents have the ability of daily living, and the proportion of urban respondents (80.08%) is slightly lower than that of rural respondents (84.09%). Most respondents in the rural area (64.57%) had a primary school and below education, while this proportion is approximately one-third (31.94%) in cities. In the resulting sample, the rate of old women was close to that of old men, and the elderly living in urban areas were more than those living in rural areas, which is close to the results of China's seventh population census. Therefore, it can be considered that our sampling results are fairly representative.

**Table 2:** Descriptive statistics of variables.

**Variable type**	**Variable name**	**Average (standard deviation)/percentage**		
		**Overall**	**Urban**	**Rural**
Dependent variable	Memory			
	Good	78.56	21.29	21.67
	Bad	21.44	78.71	73.33
Independent variable	Care for grandchildren			
	Yes	19.51	16.05	24.64
	No	80.49	83.95	75.36
	Employed			
	Yes	27.90	19.96	40.54
	No	72.10	80.04	59.46
	Re-employment service			
	Yes	4.53	2.37	7.98
	No	95.47	97.63	92.02
	Volunteer			
	Yes	24.95	29.44	20.48
	No	75.05	70.56	79.52
	Recreational activities			
	Yes	24.86	22.26	28.61
	No	75.14	77.74	71.39
	Psychological counseling			
	Yes	1.20	0.74	1.87
	No	98.80	99.26	98.13
Control variable	Gender			
	Male	45.51	41.50	51.36
	Female	54.49	42.50	48.64
	Age	75.70 (9.42)	78.05 (9.29)	72.30 (8.53)
	Disability			
	Yes	18.31	19.92	15.91
	No	81.69	80.08	84.09
	Education			
	Illiteracy	12.47	8.75	17.86
	Primary school	32.81	23.19	46.71
	Junior high school	30.67	34.03	25.82
	High school and above	24.05	34.02	9.6
	Marital status			
	Married	63.51	59.58	69.25
	Single	36.49	40.42	30.75
*N*		913	539	374

Regarding the memory of the elderly as the dependent variable, more than half of the elderly (54.63%) are unable to remember what happened within 3 days exactly. It indicating the memory of the Chinese elderly as a whole is not optimistic. It goes against the common sense of China's urban-rural differences that the memory of urban respondents is slightly lower than that of rural respondents. Through the Chi-square test, the difference in memory between urban and rural areas is not statistically significant (*p* = 0.854), so it can be considered that the phenomenon of violating common sense is caused by sampling error.

Regarding independent variables, respondents' participation in different social activities varies greatly. The most social activity that the respondents participated in was engaging in paid work after retirement, with the participation rate reaching 27.9%. However, the employment of the elderly varies greatly between urban and rural areas. The proportion of the rural elderly who still continue to work (40.54%) is significantly higher than that of the urban elderly (19.96%). Participating in volunteering or recreational activities, or taking care of grandchildren, are also social activities in which more elderly people participate, accounting for 24.95, 24.86, and 19.51%, respectively. The proportion of receiving psychocounselling was significantly less than that of other social activities with only 1% participating, which was the lowest among all social activities.

### Latent class analysis

The random seed number (851,945) appeared after the first random potential category analysis of the data by Mplus, and was used for the next fitting (the total number of samples after using the random seed number was 947). Gender was used as a predictor variable to specify a fixed random order for the data analysis, and the best fit could be obtained directly by embedding the random seed number into the subsequent fitting. Five subsequent fits with random seed numbers yielded five case categorization models.

The model fit indicators were recorded independently with statistical tests of significance for 2–6 classes (Table 3). Entropy 0.60 implies that more than 20% of instances are misclassified (<80% of cases are correctly classified), whereas Entropy >0.80 shows that more than 90% of cases are accurately categorized. The higher the Entropy score, the more realistic the model. Models 2 and 3 had an entropy of <0.70 during this data analysis, and when compared to other models, these two types of models exhibited a substantial misclassification, hence model 2 and model 3 should be eliminated. Entropy = 0.856 in model 4, suggesting that more than 90% of the instances were classified correctly. Model 6 should be rejected since its AIC, BIC, and SSBIC indices are greater than those of the other models. Model 5 is eliminated because BLRT >0.05 indicates that the number of categorical categories should be reduced.

**Table 3:** The goodness of fit of potential class models for classes 2 to 6 (*N* = 913).

**Model**	**AIC**	**BIC**	**SSBIC**	**Entropy**	**LMR**	**BLRT**
M2	4,863.277	4,965.196	4,898.502	0.667	0.0000	0.0000
M3	4,863.918	5,019.224	4,917.594	0.602	0.0167	0.0000
M4	4,878.934	5,087.626	4,951.060	0.856	0.6178	0.0000
M5	4,883.521	5,145.599	4,974.098	0.788	0.0681	0.6000
M6	4,902.355	5,217.820	5,011.384	0.813	0.6869	0.6667

According to Rost and Walter (25), classification schemes with diagonal values >0.8 are more reliable, and a higher proportion of diagonal data represent the percentage of correct classifications (14, 23). 0.037 indicates that 3.7% of secondary categories were grouped into one category. The findings in Table 4 (mean of posterior probabilities) demonstrate that Model 4 is appropriate.

**Table 4:** Means of posterior probabilities (*N* = 913).

	**1**	**2**	**3**	**4**
1	0.896	0.000	0.000	0.104
2	0.037	0.909	0.000	0.055
3	0.020	0.025	0.955	0.000
4	0.002	0.000	0.000	0.998

The underline represents the largest value in each group.

Average latent class membership probabilities (row) by latent class (Column).

In a comprehensive analysis, Model 4 is the optimal model since it more objectively reflects reality, i.e., the social engagement patterns of the elderly can be grouped into four groups.

**Figure 4:**
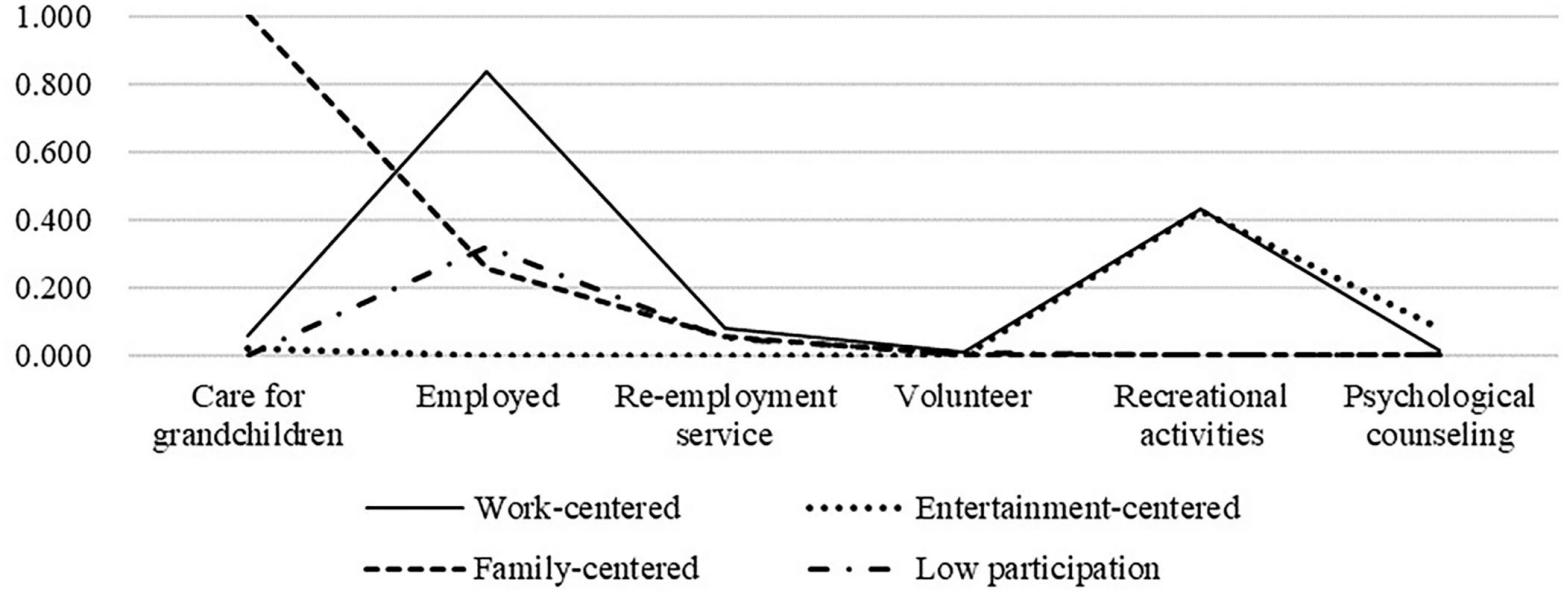
Conditional probability distribution of the four social participation patterns (*N* = 913).

As shown in Table 5 and Figure 4, the first category accounts for 10% of survey respondents. The outstanding feature of them is that they are still engaged in paid work after retirement. These seniors seem to have a high level of vitality but are identical to category 2 in terms of volunteerism, cultural engagement, and spiritual comfort. We named this pattern “work-centered.” The second category with 43.5% of the elderly, who are less active in re-employment or family participation, and more interested in cultural and sporting activities. Compared with other categories of elderly, they are more willing to receive psychological counseling services. We named this pattern “Entertainment-centered.” Third category with 18% of respondents. Most senior people in this category care for their grandchildren at home, and a few still work after retirement, but their engagement in spiritual comfort, cultural and athletic activities and volunteer services are limited. We named this pattern “Family-centered.” Low participation is classified as category 4. In addition to their required responsibilities, these elderly individuals neither care for their grandkids nor engage in recreational activities.

**Table 5:** The latent class coefficients of three-class social participation profile (*N* = 913).

**Explicit variable**	**Work-centered**	**Entertainment-centered**	**Family-centered**	**Low participation**
Care for grandchildren	0.056	0.021	1.000	0.000
Employed	0.837	0.000	0.253	0.321
Re-employment service	0.078	0.000	0.054	0.047
Volunteer	0.010	0.000	0.000	0.006
Recreational activities	0.429	0.426	0.000	0.000
Psychological counseling	0.011	0.081	0.000	0.000
Probability	0.1	0.435	0.18	0.285

To further explain the significance of the LCA findings, we'll go deeper into the characteristics of the aged in various social participation patterns. The research findings on the classification of elderly social participation reveal that family characteristics have a significant influence on elderly social participation, particularly in China (26, 27). As a result, we look at the four social engagement types from two perspectives: individual and family.

Through the χ^2^ test, we found that there were statistically significant differences in household registration, age, and family income of the elderly with four categories of social participation patterns. Most elderly with work-concern patterns live in rural and have the fewest elderly who are over 80 years old. Due to China's social security system, the pensions of most rural elderly are lower than those in urban. As a result, young rural elderly people have to continue to work to make a living. However, the rural land resources also provide conditions for them to continue their work so that their income is not low (Table 6). Most of the elderly in entertainment-centered patterns live in cities. There is the largest number of elderly people aged 80 and above among them. Although the social activities of these old people are centered on entertainment, their income is less than that of family- centered and work-centered. When only the sample group was considered, although it did not pass the χ^2^ test, the number of single in this category of elderly people was the largest among the four patterns. Because the pressure from the family is relatively low, they can enjoy their life more. Most of the elderly in the family-centered pattern are between 70 and 79 years old and living in urban, and have the highest family income among the four patterns. Living closely with family members makes them have to spend more experience taking care of their grandchildren, so they have no time to take care of entertainment and other social activities. However, the highest family income also means that their economic pressure is relatively small. The proportion of low participation elderly living in urban is the highest, while the proportion of 69 years old and below is the lowest. At the same time, their income level is also the lowest among the four patterns. These elderly people do not live with their children and are relatively difficult to obtain suitable jobs due to physical and resource constraints.

**Table 6:** Group differences in social participation of the elderly (*N* = 913).

**Variables**	**Work- centered (%)**	**Entertainment-centered (%)**	**Family-centered (%)**	**Low participation (%)**	**χ^2^**
Gender					
Male	48.39	47.62	43.87	42.19	2.334
Female	51.61	52.38	56.13	57.81	
Household registration					
Urban	45.16	59.65	56.05	64.86	11.684***
Rural	54.84	40.35	43.95	35.14	
Age					
≥80	25.81	39.36	26.11	39.00	15.987**
70–79	43.01	32.43	45.22	34.36	
≤ 69	31.18	28.22	28.26	26.64	
Family income					
Above PCDI	34.41	30.45	38.22	25.48	8.083**
Below PCDI	65.59	69.55	61.78	74.52	
Marital status					
Single	33.33	40.48	31.85	39.77	5.080
Married	66.67	59.16	68.15	60.23	
Number of children					
None	1.08	1.73	1.91	2.70	3.3388
Only one	16.13	22.77	22.29	21.62	
Two and more	82.80	75.50	75.80	75.68	
Number of children					
None	4.30	6.93	7.64	6.56	7.081
Only one	12.90	22.52	17.20	21.24	
Two and more	82.80	70.54	75.16	72.20	
Type of residence					
Not living along	91.40	92.08	89.17	92.66	1.717
Living along	8.60	7.92	10.83	7.34	
Family support					
No	65.67	68.89	74.55	60.29	6.005
Yes	34.33	31.11	25.45	39.71	

***p < 0.01.

**p < 0.05.

*p < 0.1.

It's strange that there is no significant difference in the number of children and grandchildren among the four patterns of elderly people. Although we tried to compare the family-centered with other patterns separately, the results still failed the χ^2^ test. This shows that the number of children and grandchildren does not affect whether the social participation activities of the elderly are family-centered.

For the purpose of this study, we compared four types of social participation patterns with memory, and distinguished urban and rural areas for the χ^2^ test. The result is shown in Table 7.

**Table 7:** Memory differences in the elderly with social participation patterns.

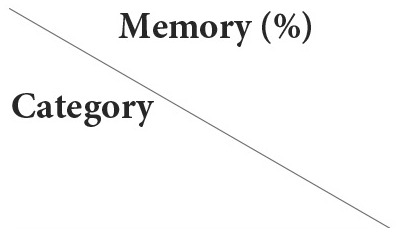	**Overall (*****N*** = **913)**		**Urban (*****N*** = **539)**		**Rural (*****N*** = **374)**	
	**Bad**	**Good**	**Bad**	**Good**	**Bad**	**Good**
Work-centered	20	80	30.77	69.23	11.76	88.24
Entertainment-centered	16.92	83.08	17.8	82.2	15.58	84.42
Family-centered	28.85	71.15	25	75	33.82	66.18
Low participation	24.4	75.6	22.09	77.91	28.74	71.26
Total	21.44	78.56	21.29	78.71	21.67	78.33
χ^2^	11.214**		4.594		14.786***	

***p < 0.01.

**p < 0.05.

*p < 0.1.

On the whole, the elderly with a good memory (80%) were significantly more than those with poor memory (20%). In comparison, the memory of the elderly in the family-centered social participation pattern is worse than others and those in entertainment-centered are the best of all. The difference passed the χ^2^ test that is statistically significant (χ^2^ = 11.214 *p* < 0.05). The situation of the urban elderly is different from the overall situation, the entertainment-centered elderly group had the largest number with good memories, while the group which had the least number with good memories is work-centered. Considering that the result of χ^2^ test fails (χ^2^ = 4.594, *p* > 0.1), these differences may also be caused by sampling errors. Finally, we compared the memory differences of the rural elderly, and the results were statistically significant (χ^2^ = 14.786, *p* < 0.01). The rural elderly with work-centered patterns of social participation has the highest percentage of good memory, and it's higher than entertainment-centered group in urban and overall. The rural elderly with family-centered patterns also has the highest percentage of bad memory in the comparison. Both in urban and rural areas, the rate of good memory of the elderly with low social participation is lower than the average (28).

It can be seen that the effects of participation in work, entertainment, and family care on memory are quite different in urban and rural areas (29). However, factors such as age and health status also play a role in social participation and memory. In order to control other related variables and conduct a more in-depth analysis, it is necessary to conduct a regression analysis.

### Logistic regression analysis

Table 8 presents the results from Logistic regression to examine the social participation pattern in the memory of the elderly. In order to reflect the role of social participation more intuitively, we take low participation as the reference group. First, we examined the effect of social participation patterns on memory in all elderly groups in Model 1. Then, in order to further study the urban-rural differences in the role of social participation, we divided the samples according to household registration and tested the effects of social participation patterns on the memory of urban and rural elderly respectively in Model 2 and Model 3. The−2log likelihood is significant in Model 1 (*p* < 0.01) and Model 3 (*p* < 0.01), indicating that they have significant explanatory power. However, Model 2 composed of urban samples failed to pass the test.

**Table 8:** Logistic regression analysis of associations between social participation pattern and memory (*N* = 913).

**Parameter**	**Model 1 (overall)**		**Model 2 (urban)**		**Model 3 (rural)**	
	**Odds ratio**	**SE**	**Odds ratio**	**SE**	**Odds ratio**	**SE**
**Social participation pattern (low participation** **=** **0)**
Work-centered	1.191	0.379	0.588	0.24	3.097**	1.708
Entertainment-centered	1.575**	0.329	1.306	0.345	2.222**	0.799
Family-centered	0.734	0.177	0.773	0.249	0.775	0.293
**Gender (male** **=** **0)**
Female	1.008	0.181	0.946	0.227	1.102	0.326
Age	0.984	0.01	0.989	0.013	0.975	0.018
**Disability (no** **=** **0)**
Yes	0.37***	0.075	0.409***	0.103	0.29***	0.103
**Education (illiteracy** **=** **0)**
Primary school	0.759	0.22	0.815	0.367	0.755	0.301
Junior High School	1.125	0.345	0.872	0.386	1.672	0.848
High School and above	0.905	0.274	0.815	0.355	0.746	0.4
**Marital status (single** **=** **0)**
Married	1.131	0.22	1.143	0.287	0.938	0.307
Constant	13.8***	13.214	12.667*	16.682	20.33*	33.901
−2 Log likelihood	−421.504***		−257.2343		−158.176***	
*R* ^2^	0.057		0.0417		0.1145	

***p < 0.01.

**p < 0.05.

*p < 0.1.

Model 1 was used to test the effect of social participation patterns on the memory of the elderly both in urban and rural. The findings indicate that compared with the pattern of low participation, only the entertainment-centered pattern (OR = 1.575, *p* < 0.05) has a significant positive effect on the memory of the elderly. This means that if other conditions are not considered, the elderly who are participated in entertainment-centered social activities is 0.575 more likely to have a good memory than those who less participate in social activities. Among the control variable, only disability is significant (OR = 0.37, *p* < 0.01). It is easy to understand that once the elderly suffered from a disability, their memory will also be negatively affected.

The results of Model 2 are against our expectations. When we only take the urban elderly as the sample, neither the logistic regression model as a whole nor the three patterns as independent variables are significant within 90% confidence. Only disability as a control variable is significant in Model 2. The results showed that for the urban elderly, the different patterns of social participation had no significant effect on their memory.

Model 3 only includes rural elderly. The result of logistic regression showed that work-centered (OR = 3.097, *p* < 0.05) and family-centered (OR = 2.222, *p* < 0.05) patterns have more positive effects on memory than low participation. Model 3 as a whole also passed the test of −2 log likelihood (−2log likelihood = −158.176, *p* < 0.001), and the *R*^2^ is 0.1145, which is higher than Model 1 (0.057) and Model 2 (0.0417). This means that model 3 can better explain the influencing factors of memory in the elderly.

## Discussion

This study divides the social participation of the elderly into four patterns: work-centered, entertainment-centered, family-centered, and low participation. By comparing and analyzing the difference among four social participation patterns by urban and rural older adults, we obtained the different influences of social participation on the memory of urban and rural older adults (30).

The result showed work-centered pattern has a significant effect on the memory of rural seniors. We also found that the memory of the elderly with this social participation pattern is very different between urban and rural areas. The proportion of urban elderly with excellent memory is lowest among those who view work as their principal social engagement. In contrast, the proportion of rural elderly with good memory is greatest among those who are still employed. We can attempt to explain this phenomenon by combining our investigational observations and the results of descriptive statistics (31). More than half of the rural elderly continue to work over 60 since their pension under the current social insurance system is insufficient to meet their basic necessities. At the same time, since the vast majority of rural people are engaged in agricultural work on their own land (70.45%), they often keep working into their later years. This means that their work content has not changed with age. Older adults who are still focused on their work keep up the same lifestyle and work routines as they had in their prime, which prevents memory loss. Due to the constraints of the retirement system in cities, older people sometimes work in fields unrelated to their past careers or even ones they have never encountered. This change in work causes them mental stress, which has a negative impact on their memory.

Compare with low participation, family-concern pattern has no significant effect on the memory of the elderly whether in urban or rural. Both urban and rural family-centered seniors have below-average memories. Especially in rural areas, the rate of a bad memory of the elderly in the family-centered model is the highest. Therefore, we can infer that family-centered social participation activities not only have no positive effects on the memory of the elderly but may even bring negative impacts. Continuing the idea that we used in the analysis of work-centered pattern, the change of roles in the family may have a negative impact on the health of the elderly. The average age of the elderly in the family-centered model is 74 years old which means they've been free from childcare for a long time. Their daily routine has been altered by caring for grandchildren, and the resulting stress has a negative impact on their memory. Although it did not pass the χ^2^ test, the number of grandchildren of the family-centered elderly was not more than others as far as the sample was concerned. They received the least family support among the four models and even had a high rate of living alone. This means that some of their grandchildren are left behind children. According to the results of our field survey, it is common in rural areas for the elderly to take care of their grandchildren while their parents work in other places. In the latent category analysis, we found that the elderly family-centered did not participate in any entertainment or volunteer activities. We can infer that their opportunities to engage in other types of social participation have been limited due to the burden of household work.

The entertainment-centered pattern significantly enhances the memory of the elderly, whether in the model with all samples or only rural samples. In urban, the elderly with an entertainment-centered pattern had the highest percentage of good memory. In general, the entertainment-centered social participation pattern has the most significant positive effect on the memory of the elderly. The main characteristic of the entertainment-centered pattern, as opposed to the work-centered pattern and the family-centered pattern, is that the elderly are responsible for the social obligations it participates in, such as those related to sports or the arts. This pleasant social activity can significantly promote the memory of the elderly. Additionally, we discovered that these elderly do not have the highest levels of family support, education, or income among the four types. This means that there are no obvious conditions for choosing the entertainment-centered social participation pattern.

The percentage of the elderly with low participation is below average. When compared to the elderly with entertainment-centered patterns and the elderly in rural areas with work-centered patterns, low participation in social activities had a detrimental effect on memory. Such old people neither take care of their grandchildren nor take part in work. Although the previous analysis suggested that the pressure brought by new responsibilities would have a negative impact on the memory of the elderly, low participation in social activities was also not conducive to the maintenance of memory. It is because they also suffer from role changes. The percentage of elderly in urban is highest with low participation patterns. Despite no additional family or career demands, retirement makes people abandon their original way of life. Without continued social interaction, these seniors' memories would deteriorate as they gradually withdraw from society (32, 33).

**Figure 5:**
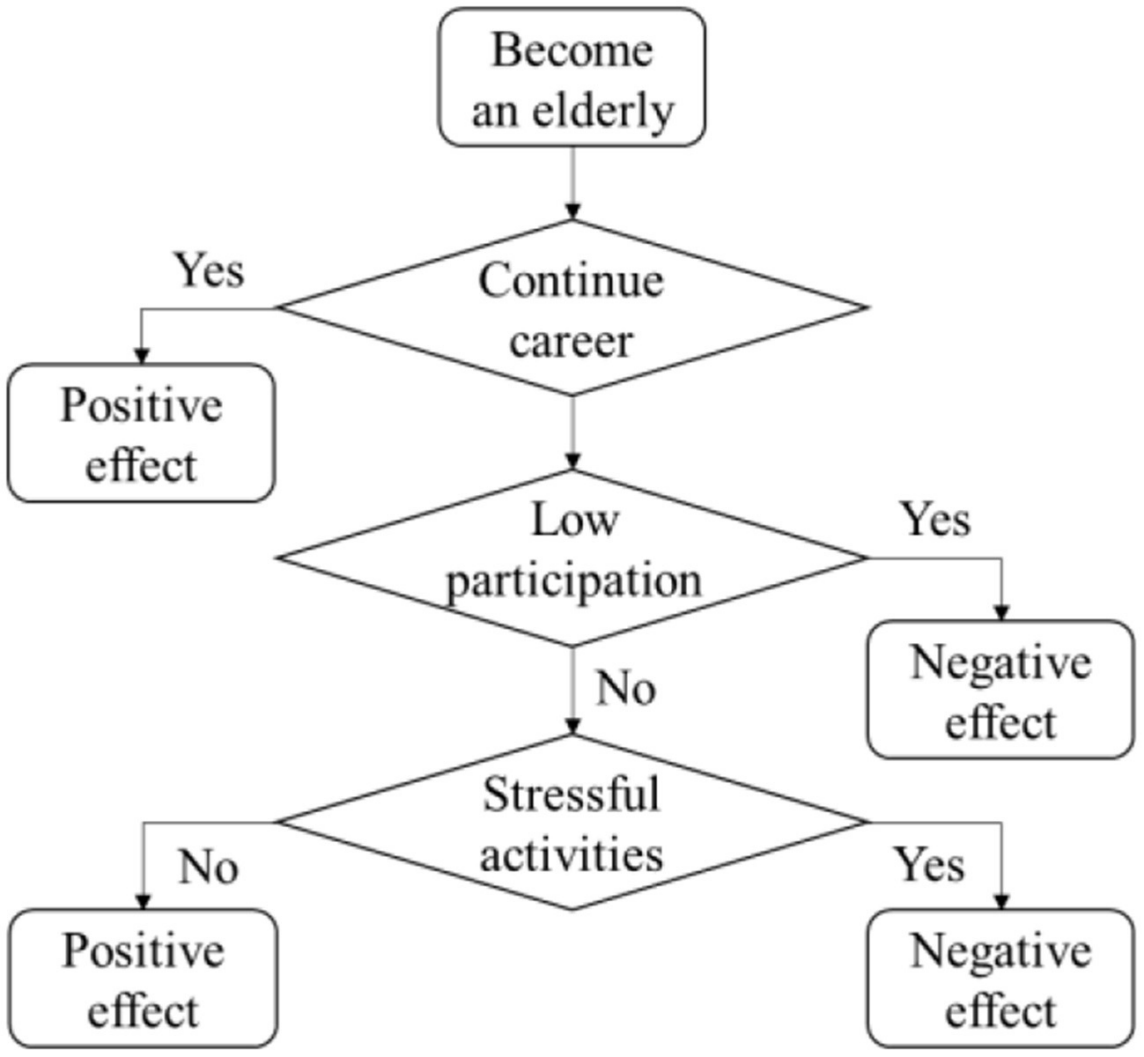
The effect of social participation on the memory of the elderly.

In summary, Figure 5 visually shows the positive or negative effects of social participation on the memory of the elderly. First of all, if the elderly continued the career which he engaged in his prime, it will have a positive impact on his memory. Next, if leaving the original living habits is unavoidable, low participation in social activities will have a negative impact on memory. Finally, for the elderly who actively participate in social activities, participation without pressure can have a positive impact on memory, while stressful social participation may have a negative impact.

## Conclusion and policy recommendations

### Conclusion

In order to understand the effect of social participation on the memory of the elderly, we divide the social participation of the elderly into four patterns through the method of latent class analysis based on the survey data from Hubei province and Zhejiang province, China. This paper compares and contrasts each of the four patterns of social participation in terms of individuals and families in order to describe the characteristics of each pattern. We investigated the fundamental causes since the logistic analysis demonstrates the impact of social participation patterns on aging memory. Our finding is strongly supported by the distinction between the social relevance of participation in urban and rural areas. The main conclusions are as follows.

First, according to personal and family spheres, this study classifies the social participation patterns of elderly people into low participation, personal entertainment-centered, personal work-centered, and family-centered through the latent class model, with proportions of 28.37, 44.25, 10.19, and 17.20% accordingly. The survey shows that entertainment-centered has the highest proportion of participants, however, these are mostly leisure activities based on community fitness equipment and chess and card rooms. A total of 30% of low participation indicate that the overall quality of social participation of elderly people in China is inadequate at present. The number of family-centered older people is higher than work-centered, which is more consistent with China's traditional family culture, where many older people devote most of their energy to family activities (21, 22, 26). The question of how much of the high participation of the elderly in the family is due to conscious taking on of family responsibilities and how much is attributable to forced choice is more deserving of our further research and attention in the context of societal transformation and changes in family structure. The rural and multi-child female elderly are more likely to be work-centered than the urban elderly. In contrast to the population distribution of low participation, the elderly with lesser income are more likely to be family-centered, whereas the younger elderly are more likely to be work-centered.

Second, after controlling demographic variables, the memory of the elderly in the entertainment-centered type was significantly better than the low participation type, and the memory of the elderly in the work-centered and entertainment-centered type in rural areas was significantly higher than low participation. This discovery provides partial support for the continuity theory. Some studies have found that the continuity of social roles is conducive to health. Due to low level of social security, rural seniors, in this study, may continue to engage in relevant labor after the age of 60 years in order to subsidize their living costs. The consistency of their life situation reduces their mental strain and emotional upheaval, which helps them remember things better. This also confirms that the simple application of “disengagement theory” or “activity theory” in the past cannot adequately explain the memory variations between urban and rural elderlies.

Third, memory variations between urban and rural elderly. The social scope of urban and rural locations is another explanation for this disparity, in addition to the continuing roles already discussed. Compared with the rural elderly, the urban elderly are more likely to benefit from the development and popularization of the Internet and smartphones. The fourth sampling survey on the living conditions of the urban and rural elderly in China in 2015 showed that 16.30% of the urban elderly owned smartphones, while this was only 5.13% for the rural elderly. Therefore, even though the low participation urban elderly can better maintain social participation through the Internet, there was no significant difference in memory between the elderly and the other three types of social participation. The rural elderly can only rely on work and entertainment activities to establish connections with the outside. Under the low participation and family mode, their social scope is greatly reduced, resulting in their memory being significantly inferior to work-centered and entertainment-centered. As a result, whether the Internet may become a new approach to increase participation, stimulate social participation of the elderly, and improve their memory is an issue that should be researched in the future (34).

### Policy recommendations

In response to the above findings, this paper proposes the following policy recommendations: firstly, for family-centered elderly people, it is necessary to recognize the value of those who provide family care, and spouses and children should take the initiative to share their responsibilities and support them, so that more elderly people can leave their homes, enrich their personal lives and enhance their physical and mental health; alternatively, there is a need to reduce the burden of family care, which requires improving the social care system for the elderly and the childcare system for 0–3-year-old, to release the extensive social participation needs of the elderly from family care (27).

Second, we should pay attention to the social participation of rural seniors. We will encourage the improvement of the rural old-age insurance system and infrastructure construction in rural regions, offering venues and facilities for rural seniors to organize and participate in social activities, and attracting more rural elderly to move out of their homes and participate in society.

Finally, steps should be implemented to encourage and enrich the social participation of urban and rural low-participation elders, as well as urban entertainment-centered seniors.

### Limitations

This study has several limitations. First, the selected independent variables cannot cover all types of social participation activities, which leads to the division of the four types of social participation modes without taking into account the differences between some important social activities. Second, most of the variables preset to describe group differences fail to pass the χ^2^ test, which makes it impossible for us to comprehensively describe the group characteristics of the four types of social participation patterns according to the research design. Thirdl, the constant items are statistically significant in the logistic regression model, which means that some factors affecting memory are not taken into account. Limited by the content of the questionnaire, our existing research could not include some factors that have been proved to have an impact on memory as control variables. Finally, the results of our study found that the internal reasons for the different effects of different patterns of social participation on the memory of the elderly are the changes in the career and the pressure caused by social participation, but our existing data could not carry out more analysis and verification of this conclusion. This requires us to do future targeted in-depth studies.

## Data availability statement

The original contributions presented in the study are included in the article/supplementary material, further inquiries can be directed to the corresponding author.

## Ethics statement

The studies involving human participants were reviewed and approved by the medical Ethics Committee of the Health Science Center of Xi'an Jiaotong University (Approval Number 2018–1200). The participants provided their written informed consent to participate in this study.

## Author contributions

HH designed the study, constructed the analytical framework, participated in the statistical analysis, and drafted the manuscript. ZH performed the data calculation and statistical analysis and revised the manuscript. TY organized the survey and revised the manuscript. All authors have approved the final version for publication.

## Funding

This study was funded by Key Grant Project of Chinese Ministry of Education (No. 18JZD045), Chinese Postdoctoral Science Foundation (No. 2019M663773), Projects on Major Theoretical and Practical Issues in Philosophy and Social Sciences (No. 2022ND0311), and Key Projects of Shaanxi Provincial Education Department (No. 22JZ042).

## Conflict of interest

The authors declare that the research was conducted in the absence of any commercial or financial relationships that could be construed as a potential conflict of interest.

## Publisher's note

All claims expressed in this article are solely those of the authors and do not necessarily represent those of their affiliated organizations, or those of the publisher, the editors and the reviewers. Any product that may be evaluated in this article, or claim that may be made by its manufacturer, is not guaranteed or endorsed by the publisher.
